# Changes in Intestinal Microbiota Are Associated with Islet Function in a Mouse Model of Dietary Vitamin A Deficiency

**DOI:** 10.1155/2020/2354108

**Published:** 2020-01-21

**Authors:** Yunting Zhou, Junming Zhou, Yumin Zhang, Jun Tang, Bo Sun, Wei Xu, Xiaohang Wang, Yang Chen, Zilin Sun

**Affiliations:** ^1^Department of Endocrinology, Zhongda Hospital, Institute of Diabetes, School of Medicine, Southeast University, Nanjing, China; ^2^Department of Gastroenterology, Jinling Hospital, Medical School of Nanjing University, Nanjing, China; ^3^Department of Anesthesiology, Jinling Hospital, Medical School of Nanjing University, Nanjing, China; ^4^State Key Laboratory of Bioelectronics, School of Biological Science and Medical Engineering, Southeast University, Nanjing, China; ^5^Department of Diabetes, School of Life Course Sciences, King's College London, Guy's Campus, London, UK

## Abstract

**Aims:**

The underlying mechanisms involved in Vitamin A- (VA-) related changes in glucose metabolic disorders remain unclear. Recent evidence suggests that intestinal microbiota is closely linked to the metabolic syndrome. Here, we explored whether and how intestinal microbiota affects glucose homeostasis in VA-deficient diet-fed mice.

**Methods:**

Six-week-old male C57BL/6 mice were randomly placed on either a VA-sufficient (VAS) or VA-deficient (VAD) diet for 10 weeks. Subsequently, a subclass of the VAD diet-fed mice was switched to a VA-deficient rescued (VADR) diet for an additional 8 weeks. The glucose metabolic phenotypes of the mice were assessed using glucose tolerance tests and immunohistochemistry staining. Changes in intestinal microbiota were assessed using 16S gene sequencing. The intestinal morphology, intestinal permeability, and inflammatory response activation signaling pathway were assessed using histological staining, western blots, quantitative-PCR, and enzyme-linked immunosorbent assays.

**Results:**

VAD diet-fed mice displayed reduction of tissue VA levels, increased area under the curve (AUC) of glucose challenge, reduced glucose-stimulated insulin secretion, and loss of *β* cell mass. Redundancy analysis showed intestinal microbiota diversity was significantly associated with AUC of glucose challenge and *β* cell mass. Redundancy analysis showed intestinal microbiota diversity was significantly associated with AUC of glucose challenge and *κ*B signaling pathway activation. Reintroduction of dietary VA to VAD diet-fed mice restored tissue VA levels, endocrine hormone profiles, and inflammatory response, which are similar to those observed following VAS-controlled changes in intestinal microbiota.

**Conclusions:**

We found intestinal microbiota effect islet function via controlling intestinal inflammatory phenotype in VAD diet-fed mice. Intestinal microbiota influences could be considered as an additional mechanism for the effect of endocrine function in a VAD diet-driven mouse model.

## 1. Introduction

Vitamin A deficiency (VAD) poses a serious threat to public health in developing countries [1]. VA and its retinoid metabolism pathway play a central role in maintaining the functions of pancreatic endocrine *β* cells and peripheral insulin sensitivity in the adult pancreas [2, 3]. Several mechanistic studies show that VAD induces endoplasmic reticulum stress [4], causes apoptosis in pancreatic islet cells [5], inhibits activation of the insulin signaling cascade in insulin-sensitive tissues [6], and limits hepatic glucokinase activity of hepatic glucose metabolism [7]. Although many studies have investigated the molecular basis of VAD-associated glucose disorders, the exact pathogenic mechanisms involved remain unknown.

Intestinal microbiota is recently referred as a “hidden organ,” including a wide range of bacteria, with an extension of a gene pool much more abundant than that from the host. Intestinal microbiota and perturbations in the composition of the microbiota support numerous nutritional, metabolic, immunological, and physiological processes [8–11]. Amit-Romach et al. [12] found that VAD diets alter the composition of intestinal microflora by decreasing the relative proportion of lactobacillus spp. and total number of bacteria in the gastrointestinal tract, and damaging the integrity of the gastrointestinal mucosal barrier. The diversity of intestinal microbiota and important phylotypes significantly differed in children with persistent diarrhea at different VA nutritional levels. Sequencing of fecal microbiota indicates that VAD leads to a reduction in the diversity of microbiota involved in the remodeling of opportunistic pathogens and butyrate-producing bacteria [13]. Thus, the intestinal microbiome with functional and compositional shifts may help us to identify new mechanisms that explain the occurrence and progression of diseases in host metabolism.

To date, the mechanisms by which intestinal microbiota affect VAD-related glucose metabolic disorders have not been proposed. Therefore, the aim of this study was to test the effects of VA on glucose homeostasis and determine the relationship between changes in intestinal microbiota and VAD-driven islet dysfunction using a VA-deficient diet-induced mouse model. We also determined how VA-driven changes in intestinal microbiota affect endocrine dysfunction, thereby exploring a novel therapeutic strategy for VAD-driven pancreatic impairment through intestinal microbiota modulation.

## 2. Methods

### 2.1. Animals and Diet

Six-week-old male C57BL/6 (*n* = 10/group) mice were purchased from the Model Animal Institute of Nanjing University. The animals were bred in a controlled environment (12 h day/light cycle) with food and water provided *ad libitum*. Model mice were conducted according to the previously described method in [5] with some modifications. We bought VA-deficient purified (VAD, VA < 120 IU/kg), VA-control sufficient purified (VAS, 15,000 IU/kg VA), and VA-deficient rescued purified diets (VADR, 35,000 IU/kg VA) from Animal Diets Co., Ltd. (Changzhou, China). The mice were randomly separated into experimental groups, VAD and VAS groups. For the VAD group, mice were deprived of VA for 10 weeks. For the VADR group, a subgroup of the VAD mice was then placed on a VA-excess sufficient purified diet for further 8 weeks. The tissue and serum samples were collected in the dark and stored at -80°C until use. Random blood glucose measurements were taken at two or three random time points weekly over the duration of the experiment. All experimental procedures were conducted in accordance with the National Institutes of Health Guide for the Care and Use of Laboratory Animals (NIH Publications No. 8023, revised 1978).

### 2.2. Intraperitoneal Glucose Tolerance Test (IPGTT)

Blood samples were obtained from the tail veins of mice that had been fasted for 8 h, at 0, 15, 30, 60, and 120 min following intraperitoneal injection of glucose (2 g D-glucose.kg^−1^). Blood glucose levels were monitored at 0, 15, 30, 60, and 120 min following injection using a glucose monitor (Bayer, Geneva, Switzerland). Areas under the curve (AUC) for the blood glucose-time function, such as AUC_IPGTT-glucose_ and AUC_IPGTT-insulin_, were calculated using SigmaPlot software (Systat Software, San Jose, CA, USA).

### 2.3. Oil Red O Staining

Frozen tissue sections were fixed in 4% paraformaldehyde (PFA) for 20 min at room temperature and then rinsed with 60% isopropanol for 5 min. Subsequently, the sections were stained with freshly prepared Oil red O working solution (3 : 2 of 0.5% Oil red O stocking solution/distilled water) for 20 min at room temperature. The samples were washed with distilled water and observed using phase contrast microscopy (Zeiss, Oberkochen, Germany).

### 2.4. 16S Gene Sequencing

We extracted DNA from caecal content of randomly selected male mice (6 per group) using the QIAamp® DNA Stool Mini Kit (Qiagen, Hilden, Germany) as per the manufacturer's instructions. DNA was quantified using the Qubit quantification system (Thermo Scientific, Wilmington, DE, US). The V3-V4 hypervariable region of the 16S rRNA gene was amplified from genomic DNA using the following primers: 341F (CCTACGGGNGGCWGCAG) and 805R (GACTACHVGGGTATCTAATCC). The 16S rRNA gene amplification was performed using a mixture of the KAPA HiFi Hot start Ready Mix, 0.1 *μ*M of primer 341 forward, and 0.1 *μ*M of primer 805 reverse in 96-well microtiter plates. A total of 12.5 ng DNA was present in a 50 *μ*l volume of sample. Reactions were running in a T100 PCR thermocycle (BIO-RAD) under the following conditions: 94°C for 3 min, followed by 18 cycles of 94°C for 30 s, 55°C for 30 s, and 72°C for 30 s, with a final extension at 72°C for 5 min. The amplicons were checked and quantified using the Qubit quantification system (Thermo Scientific, Wilmington, DE, US) following the manufacturers' instructions. Next, 2 *μ*l of the diluted amplicons were mixed with a reaction solution containing sequencing primers and adaptors consisting of 1× KAPA HiFi Hotstart ReadyMix, 0.5 *μ*M of fusion forward and reverse primers, and 30 ng of meta-gDNA in a 50 *μ*l volume. The amplification products were purified and quantified according to the manufacturer's instructions. The concentration of the pooled libraries was determined using the Qubit quantification system. Amplicon sequencing was subsequently performed on the Illumina MiSeq System (Illumina Inc., CA, USA). Automated cluster generation and paired-end sequencing were performed using a composite of dual-index reads.

### 2.5. High-Performance Liquid Chromatography (HPLC)

The frozen tissue samples from the pancreas, intestines, and liver (100-200 mg) were minced into small pieces and rinsed in ice-cold PBS (phosphate-buffered saline) to remove excess blood thoroughly. Then tissue pieces were homogenized in PBS (tissue weight (g) : PBS (ml) volume = 1 : 1) in glass homogenizers. The volumes were adjusted to 500 *μ*l with PBS. The retinoid was extracted into 350 *μ*l of organic solution (acetonitrile/butanol, 50 : 50, *v*/*v*) in the dark. Retinoid samples (serum and tissues) were measured by a Waters Alliance HPLC analyzer (Waters 2695, USA) at Shanghai Adicon Clinical Laboratories. The levels of tissue retinol were normalized to the tissue weight.

### 2.6. Enzyme-Linked Immunosorbent Assay (ELISA)

Mouse VA (retinol) levels in serum were measured using an ultrasensitive mouse-specific ELISA kit (NBP2-60192, Novus, USA). Mouse serum insulin levels, plasma lipopolysaccharide (LPS), and plasma tumor necrosis factor-*α* (TNF-*α*) concentrations from each sample were measured using a mouse-specific ELISA kit as specified (both from MeilianBio, China). Mouse I*κ*B Kinase (IKK) levels in intestinal tissues were measured using a mouse-specific ELISA kit (MeiMianBio, China).

### 2.7. Histology and Immunocytochemistry

Paraffin sections of pancreatic, liver, and intestinal tissues from each sample were stained with hematoxylin and eosin (H & E) for histological examination using a standard protocol [14]. Frozen sections (8 *μ*m thickness) were fixed with 4% PFA and blocked with 0.2% Triton X-100 and 5% bovine serum albumin (BSA) for 30 min each at room temperature. The sections were then incubated with the target protein antibody, rabbit anti-mouse insulin (Cell Signaling Technology, USA, 1 : 200 dilution), followed by secondary antibody, Alexa Fluor 488-conjugated donkey anti-rabbit immunoglobulin G (IgG, 1 : 300, Abcam), and 4′,6′-diamidino-2-phenylindole was used for nuclei staining. For measurements of the islet area and *β* cell mass, islets in immunocytochemistry sections from each mouse were identified from every serial section. The mean islet area in each section was then calculated using Image-Pro software (Media Cybernetics, USA).

### 2.8. Quantitative PCR (q-PCR)

Total RNA of tissues per sample were extracted using TRIzol and 2 *μ*g RNA were reverse-transcribed using 5× All-In-One MasterMix (Abcam, Canada) in a PCR iCycler (Thermo, USA) according to the manufacturer's instructions. SYBR® Green Ex Taq™ II mix (Takara, Japan) was used for q-PCR performance. PCR was performed via the Step One Real-Time PCR System using the following conditions: 95°C for 30 s, followed by 40 cycles of 95°C for 5 s and 60°C for 30 s (Applied Biosystems, CA, USA). Specific primers were designed using sequences in the GenBank database, which are listed in Table 1.

### 2.9. Western Blotting

Proteins extracted from tissues were homogenized using a radioimmunoprecipitation assay buffer consisting of phenylmethylsulfonyl fluoride and phosphatase inhibitor cocktail. After protein was quantified using a BCA assay (Thermo Scientific, USA), 20 *μ*g of protein samples were separated using 10% sodium dodecyl sulfate polyacrylamide gel electrophoresis and transferred to nitrocellulose membranes. Then, the following primary antibodies were used: rabbit anti-Toll like receptor 4 (TLR4) (Abcam, USA; 1 : 1000 dilution), rabbit antimyeloid differentiation factor 88 (MyD88), rabbit antinuclear factor-*κ*B (NF-*κ*B) (Cell Signaling Technology, USA, 1 : 1000 dilution), and mouse anti-*β*-actin (ZS-Bio, China; 1 : 3000 dilution). Following this, proteins were washed using tris-buffered saline buffer (5% Tween-20), and secondary antibodies were incubated. The density of the bands was visualized using a chemiluminescent substrate kit (Thermo Scientific, USA). Quantification analyses were performed using ImageJ software.

### 2.10. Statistical Analysis

The OTUs (operational taxonomic units) were analyzed using phylogenies in the Quantitative Insights into Microbial Ecology (QIIME) software (Version 1.9.0). The results were analyzed using *t*-test, one-way or two-way ANOVA analyses; multiple correlation analyses were assessed by Pearson's test of variance using GraphPad Inc. software (Version 5.00) and expressed as mean ± S.D. Statistical significance was set at *p* < 0.05 according to *post hoc analyses*.

## 3. Results

### 3.1. VAD Causes Reduction of Tissue VA Levels

**Figure 1:**
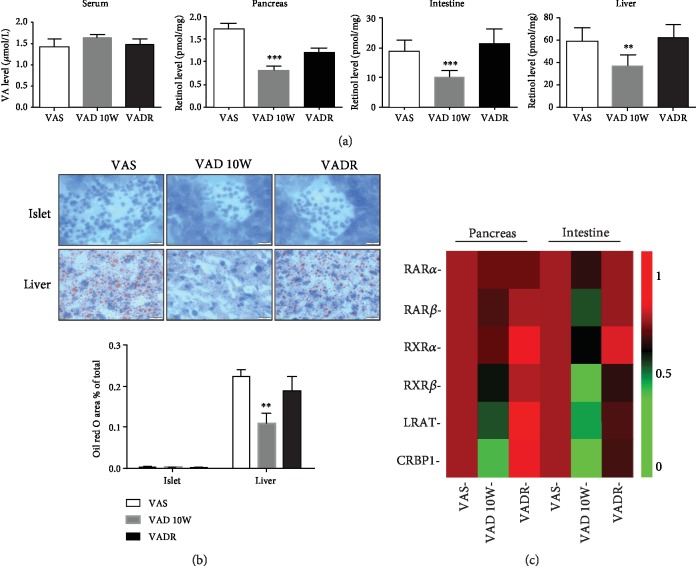
VAD leads to reduction of tissue VA levels. (a) VA (retinol) levels in the serum, pancreas, intestines, and liver of mice from VAS, VAD, and VADR groups. (b) Representative photomicrographs of lipid droplets in the liver and islets using Oil red O staining from mice described for (a). (c) Heat map of PCR measurements of relative pancreatic/intestinal mRNA levels involved in VA metabolic signaling from mice described for (a). Magnification: 100x; scale bars, 20 *μ*m. Error bars represent S.E. ^∗^*p* < 0.05, ^∗∗^*p* < 0.01, and ^∗∗∗^*p* < 0.001.

After 10 weeks of VA diet deprivation, there were no significant changes in serum VA (retinol) levels detectable in all groups. However, pancreatic, intestinal, and liver VA (retinol) levels were 53%, 47%, and 37% lower in the VAD group than those in the VAS group, respectively. After the VADR diet treatment, pancreatic, intestinal, and liver VA (retinol) levels were restored to levels similar to those in the VAS group (Figure 1(a)). Considering the liver is the major tissue site of retinol storage in the body, lipid-droplet levels from this retinoid store are also needed to quantitatively support the VAD model construction [15]. Oil red O staining revealed there was a decreased lipid deposition in the liver of the VAD group, as evidenced by the smaller size and lower abundance of lipid droplets. However, this was not observed in the islets (Figure 1(b)). Furthermore, we also determined relative mRNA levels of retinoid receptors, enzymes, and binding protein carriers in pancreatic and intestinal tissues. Quantitative PCR analysis of tissues from mice of VAD and VADR groups showed that the mRNA levels of retinoic acid receptor *α* (RAR*α*) remained unaffected in the pancreas and intestine. In contrast, the mRNA levels of retinoic acid receptor *β* (RAR*β*), retinoid X receptor *α* (RXR*α*), retinoid X receptor *β* (RXR*β*), and lecithin retinol acyltransferase (LRAT), especially intracellular retinol binding protein 1 (CRBP1), were greatly reduced in pancreatic and intestinal tissues from VAD diet-fed mice compared with those from VAS diet-fed mice (Figure 1(c)).

### 3.2. VAD Alters Islet Morphology, Decreased *β* Cell Mass, and Impaired Glycemic Responses

**Figure 2:**
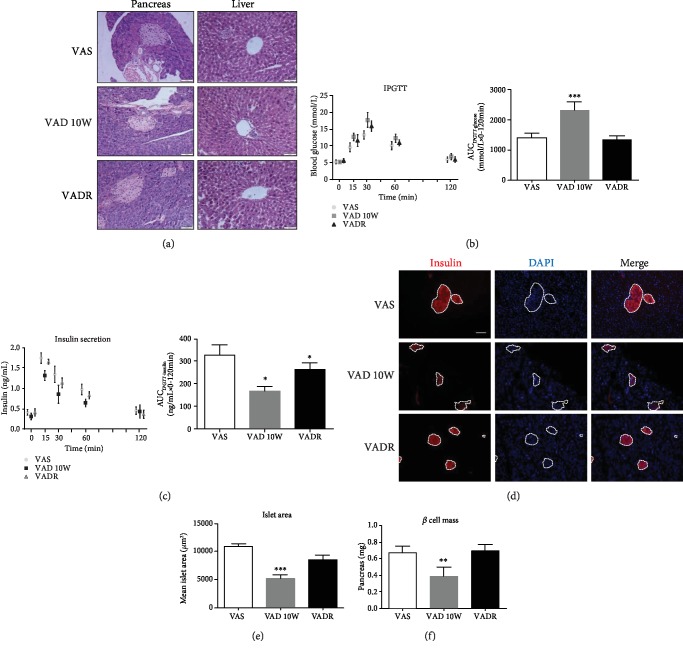
VAD leads to irregular islet morphology, loss of *β* cell mass, and impaired glycemic responses. (a) H & E staining of pancreatic islet and liver sections of mice from VAS, VAD, and VADR groups. Magnification, 40x; scale bars, 50 *μ*m. (b, c) Blood glucose and insulin levels using the IPGTT test were analyzed of mice described for (a). The results of the IPGTT test were analyzed by AUC (i.e., AUC_IPGTT-glucose_ or AUC_IPGTT-insulin_). (d) Representative immunofluorescence images of pancreatic islet sections of mice described for (a) stained with antibodies against insulin. Magnification: 20x; scale bars, 100 *μ*m. (e) Mean islet area (*μ*m^2^) of pancreas of mice described for (a). (f) *β* cell mass of mice described for (a). Error bars represent S.E. ^∗^*p* < 0.05, ^∗∗^*p* < 0.01, and ^∗∗∗^*p* < 0.001.

Pancreatic sections stained with H & E revealed changes of islet architecture, such as irregularly shaped islet outlines, in VAD diet-fed mice compared with those of VAS diet-fed mice. Unlike pancreatic tissues, the histology of parenchyma cells in the liver of VAD diet-fed mice was not altered (Figure 2(a)). After 10 weeks on VAD diet, blood glucose levels at 15, 30, and 60 min were higher than those of the control mice in the abilities of the glucose response using IPGTT. For glucose-stimulated insulin secretion, AUC_IPGTT-insulin_ decreased in VAD diet-fed mice than that in VAS diet-fed mice. Therefore, peripheral insulin sensitivity was lower in VAD diet-fed mice than that in the controls as evidenced by an increased AUC_IPGTT-glucose_ (Figures 2(b) and 2(c)). However, random blood and fasting blood glucose levels were similar in all treatment groups (data not shown). Furthermore, immunohistochemistry assay showed decreased insulin signal in the islets of VAD diet-fed mice; however, the VADR diet-fed mice normalized pancreatic insulin signal (Figure 2(d)). Measurements of the islet morphology by direct morphometric and mathematical model analyses showed that dietary VA deprivation led to a significantly lower mean pancreatic islet area and *β* cell mass than those of control mice, consistent with the frequent appearances of smaller islet clusters in the population. In VADR diet-fed mice, we did not observe smaller islet clusters and loss of *β* cell mass (Figures 2(e) and 2(f)).

### 3.3. VAD Induces Changes in Intestinal Microbiota Structure, Richness, and Community Difference

**Figure 3:**
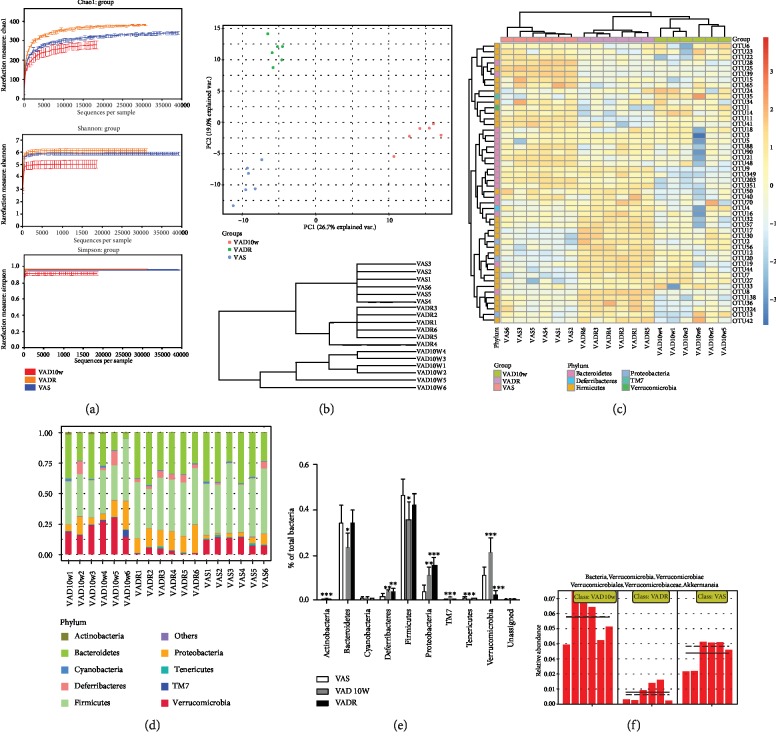
VAD causes reduced diversity/richness and alters cluster and composition of intestinal microbiota. (a) Alpha-diversity assay of intestinal microbiota of mice from VAS, VAD, and VADR groups. (b) Beta-diversity of intestinal microbiota of mice described for (a): unweighted UniFrac PCoA plotted against PC1 versus PC2 axes. (c) Cluster analysis for bacterial communities of mice described in (a). (c) Heat map of key OTUs of mice described for (a). (d, e) Bacterial composition at the phylum level of mice described for (a). (f) Akkermansia muciniphila composition at the phylum level of mice described for (a). Error bars represent S.E (*n* = 6/group). ^∗^*p* < 0.05, ^∗∗^*p* < 0.01, and ^∗∗∗^*p* < 0.001.

Across six samples in each group, 635,743 valid sequences and 5,618 OTUs were retrieved. The mean length of these sequences was 378 bp. Based on Chao, Shannon, and Simpson reaction estimators, the diversity and richness of the intestinal microbiota were significantly lower in VAD diet-fed mice than those in VAS diet-fed mice (Figure 3(a)). Beta-diversity using principal coordinate analysis (PCoA) revealed the divergence of bacterial community structure of VAD diet-fed mice from the VADR diet-fed mice and VAS diet-fed mice, with PC1 accounting for 26.7% of the total variation. Cluster analysis using an unweighted UniFrac method also showed bacterial communities in the VADR diet-fed mice and VAS diet-fed mice were clustered closely to each other, while the communities were clustered alone in the VAD diet-fed mice (Figure 3(b)). The heat map of the top 50 key OTUs in three groups is shown in Figure 3(c). Compared with the VADR diet-fed mice and VAS diet-fed mice, 23 OTUs had a significant difference in VAD diet-fed mice based on the analysis of Euclidean distance. We also compared the community difference in microbiota at phylum, class, order, family, and genus level to explore the overall variation in intestinal microbiota composition among the three groups. A total of 10 phyla were identified; Bacteroidetes, Firmicutes, Proteobacteria, and Verrucomicrobia were identified as the four major phyla (>0.1% of total composition). Tenericutes, Actinobacteria, Cyanobacteria, TM7, and other unclassified bacteria were identified as the minority phyla. Ratios of Firmicutes to Bacteroidetes (F/B) in VAD diet-fed mice, VAS diet-fed mice, and VADR diet-fed mice were 0.71, 0.65, and 0.78, respectively. The VAD diet-fed mice contained a lower abundance of Bacteroidetes, but higher abundance of Firmicutes than that in VAS diet-fed mice. Additionally, the abundance of Bacteroidetes in the VADR diet-fed or VAS diet-fed mice was 0.10% and 0.07%, respectively, higher than that in the VAD diet-fed mice (Figures 3(d) and 3(e)). Furthermore, at a Verrucomicrobia class level, there was a significant increase in Akkermansia muciniphila in the VAD diet-fed mice, but a decrease in VADR diet-fed mice, compared with VAS diet-fed mice (Figure 3(f)).

### 3.4. VAD-Driven Intestinal Microbiota Changes Are Associated with Islet Function

**Figure 4:**
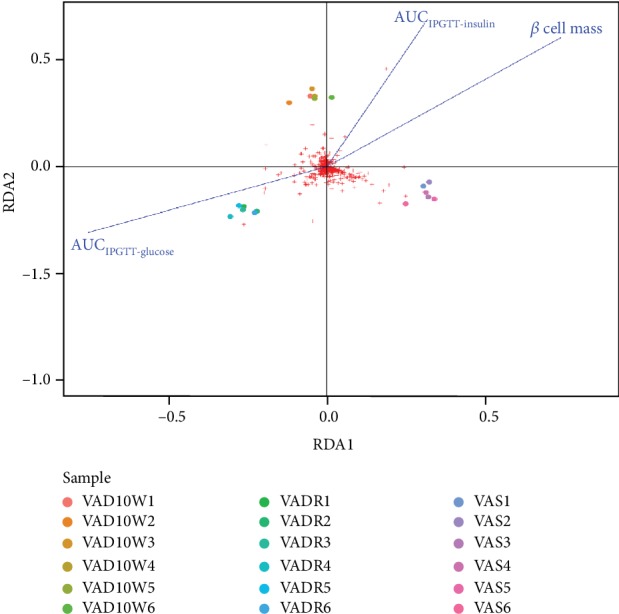
Correlation analysis of changes in intestinal microbiota and biochemical parameters of islet function. Redundancy analysis of the significant relationship between islet function and bacterial community of VAS, VAD, and VADR groups (*n* = 6/group).

To identify whether changes in VAD diet-driven intestinal microbiota are associated with islet function, multiple correlation analyses between these parameters were performed. In the redundancy analysis biplot, five significant biochemical variables were selected, AUC_IPGTT-glucose_, AUC_IPGTT-insulin_, *β* cell mass, fasting glucose levels, and random glucose levels. The AUC_IPGTT-glucose_, AUC_IPGTT-insulin_, and *β* cell mass appeared to be the more important parameters, whereas we found no significant correlation between the other parameters (data not shown). A negative correlation between AUC_IPGTT-glucose_ and bacterial community was found in mice a VAD fed, but a positive correlation between AUC_IPGTT-insulin_ and *β* cell mass and bacterial community was found (Figure 4).

### 3.5. VAD-Driven Intestinal Microbiota Changes Increase Systemic and Intestinal Inflammatory Response

**Figure 5:**
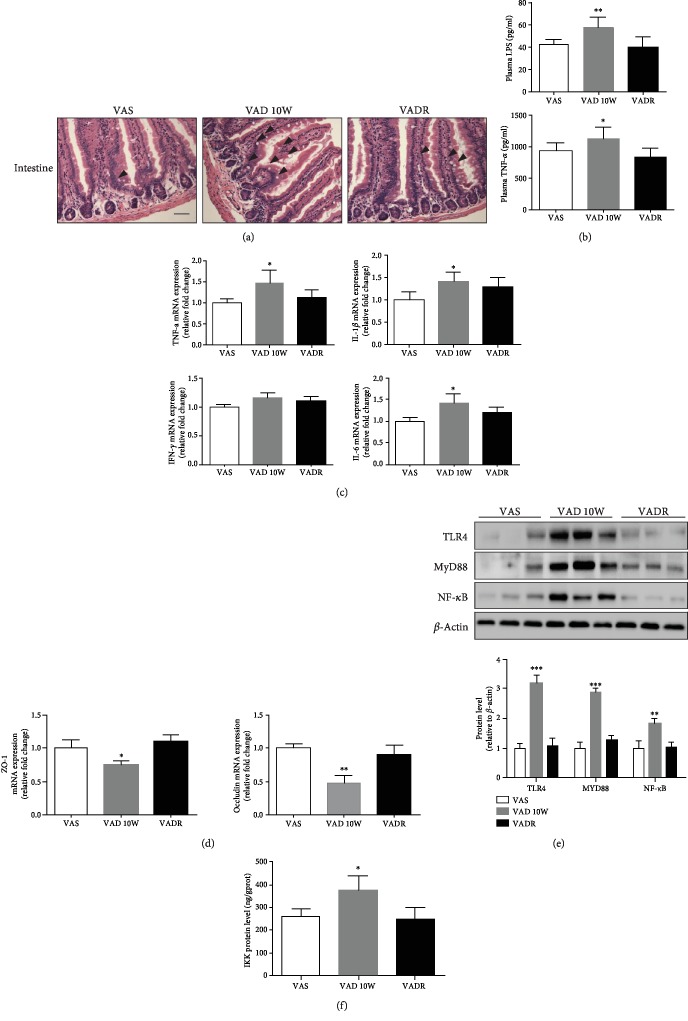
VAD induces inflammatory responses in the intestine by activating the NF-*κ*B signaling pathway. (a) Representative photomicrographs of intestinal sections of mice from VAS, VAD, and VADR groups using H & E staining. (b) Plasma LPS and TNF-*α* concentrations were analyzed of mice described for (a) by ELISA. (c, d) PCR measurements of relative intestinal inflammation markers (TNF-*α*, IFN-*γ*, IL-6, and IL-1*β*) and tight-junction integrity markers (ZO-1, occludin) of mice described for (a). (e) The protein levels in NF-*κ*B signal pathways were analyzed of mice described for (a) using western blotting. (f) The protein levels of IKK were analyzed of mice described for (a) using ELISA. Error bars represent S.E (*n* = 6/group). ^∗^*p* < 0.05, ^∗∗^*p* < 0.01, and ^∗∗∗^*p* < 0.001.

Intestinal morphological analysis by H & E staining showed intestinal tissues from VAD diet-fed mice were partly short and disordered compared with those from VAS diet-fed mice, which were well-developed, tall, and thin. There was a tendency toward crypt elongation in VAD diet-fed mice, although there was no significant difference among the three groups (Figure 5(a)). The systemic plasma LPS and TNF-*α* concentrations increased to 58.2 pg/ml (1.3-fold) and 1245.8 pg/ml (1.2-fold) in VAD diet-fed mice compared to those of VAS diet-fed mice, respectively (Figure 5(b)). The inflammatory cytokine levels in intestinal tissues, such as TNF-*α*, interferon-*γ* (IFN-*γ*), interleukin-6 (IL-6), and interleukin-1*β* (IL-1*β*), were profiled using q-PCR assay. The mRNA levels of TNF-*α*, IL-6, and IL-1*β*, apart from IFN-*γ* were significantly higher in VAD diet-fed mice than those in VAS diet-fed mice (Figure 5(c)). Simultaneously, the mRNA levels of zona occludens-1 (ZO-1) and occludin, the key markers of tight-junction integrity [16], were lower in the intestinal segment from VAD diet-fed mice than those from VAS diet-fed mice (Figure 5(d)). The abundance of inflammatory cytokines in intestines from VAD diet-fed mice prompted us to assess the activation state of the classic inflammatory signaling pathway TLR4-NF-*κ*B in the intestine. We found a 1.8-fold elevation of NF-*κ*B and a 3.2- and 2.8-fold elevation of the upstream proinflammatory effectors of the NF-*κ*B signaling pathway, TLR-4 and MyD88, respectively, in VAD diet-fed mice compared with those in VAS diet-fed mice (Figure 5(e)). Similarly, the IKK levels increased by 43% in VAD diet-fed mice using the ELISA assay (Figure 5(f)). The data demonstrated that VAD diet-driven intestinal microbiota modifies the inflammatory response by activating the NF-*κ*B signaling pathway.

## 4. Discussion

In our present study, the experimental model was established using VA-sufficient diet as the control group and the rescued purified diet for rodents of VAD diet-fed mice contains excess levels of VA for the VADR group. The model differs from the spontaneous, transgenic modified model, in which the islet microenvironment is the salient pathological feature [5, 17–19]. Thus, the diet-induced model used here was an ideal model that allowed us to better analyze the effects of VA on endocrine function *in vivo* because it is a better representation of the occurrence of VA in everyday circumstances. Serum VA (retinol) levels are under tight hepatic homeostatic control and do not decline until VA (retinol) concentration in the liver is almost depleted. In other words, serum VA (retinol) levels only changes in severe VA deficiency [20, 21]. Mice deprived of VA at 3 weeks postpartum display normal serum retinol concentrations for up to 20 weeks [22]. In our study, changes in serum VA (retinol) levels were not detected, but there are significant reductions in pancreatic, liver, and intestinal VA (retinol) levels in the VAD group compared with the VAS group. It is possible that this only occurred in the organs of VAD diet-fed mice because they had impaired VA metabolic signaling before serum VA (retinol) levels have changed, including reductions in retinoic acid receptor and intracellular retinoid metabolic enzyme and carrier proteins. This finding is also consistent with Trasino et al.'s studies [23] which found reductions in VA (retinol) levels and the gene expression of VA metabolic signaling in multiple organs but found no changes in serum VA levels in obese mice.

Our IPGTT tests showed that the glucose metabolic phenotype was damaged with increased relative severity of impaired glucose tolerance in VAD diet-fed mice. Impairment of islet architecture, decreased islet insulin fluorescence intensity, and reduction of mean islet area in VAD diet-fed mice further suggested that VA altered the degree of glucose intolerance, which correlated with islet structure and *β* cell mass. However, without evidence of changes in random and fasting blood glucose levels, we considered that the pancreas may adapt other mechanisms to maintain its normal secretory function in response to nutrient deficiency and rescue conditions. The importance of the influence of intestinal microbiota on the exocrine and endocrine pancreas is becoming increasingly clear [24, 25]. Our study is based on the theory that intestinal microbiota changes induced by VA may contribute to changes in the pancreas/glucose metabolism, particularly in the capacity of the pancreas to maintain glucose homeostasis.

Based on OUT, cluster, diversity, and richness analyses of the structure of the intestinal microbiota, we found that VAD effectively inhibited the sequence number and OTU due to the loss of intestinal microbiota, resulting in lower numbers of phymals. However, VA resumption therapy restored the structure of the microbiota to normal levels as seen in the VAS group. Although individuals have vastly different intestinal microbiomes, intestinal microbiota contained main members of four phyla (Firmicutes, Bacteroidetes, Actinobacteria, and Proteobacteria) [26]. Further analysis showed that VAD resulted in the disproportionate abundances of Firmicutes and Bacteroidetes in the intestines. Bacteroidetes and Firmicutes are basically composed of gram-negative bacteria, in addition to the gram-positive Firmicutes microorganisms [27, 28], and several kinds of polysaccharide degrading enzymes can be produced by the Bacteroidetes phylum, while the present number of microbes with the capacity of polysaccharide degradation was fewer in the Firmicutes phylum [29, 30]. Thus, VAD could lead to intestinal microbiota dysbiosis, a condition where microbial imbalance exerts adverse effects on the host [31], which might result in VAD-driven intestinal microbiota structure changes correlated with biochemical markers of pancreatic endocrine function. Interestingly, we also detected a higher proportion (>0.05%) of the mucus-degrading genus Akkermansia, belonging to the phylum Verrucomicrobia, in VAD diet-fed mice. It has been proposed that the growth of this bacterium is favored by low availability of enteral nutrients such as in long-term fasting and malnutrition [32]. However, some agree that this genus is a potential biomarker of a healthy gut status [33]. Recent data have shown that the abundance of Akkermansia muciniphila is dramatically decreased in obese and type 2 diabetes patients. Akkermansia muciniphila treatment can reverse high-fat diet-induced metabolic disorders, including fat-mass gain metabolic endotoxemia, adiposity, and insulin resistance [34, 35]. In our study, Akkermansia muciniphila was not contributing in VAD diet-fed mice. We considered that it may be a compensatory increase in intestinal flora when inflammatory response is elevated. Owing to the more effective characterization of the metatranscriptome or metabolome at the functional level, further work is necessary to explore more detailed functional hierarchy and clarify the effects of these changes in intestinal flora using metagenomic analysis.

Low-grade metabolic inflammation is one of the more important characteristics of metabolic syndrome and diabetes [36, 37]. Our result showed there were an increase in mRNA expression of inflammatory factors and intestinal permeability in intestinal tissues of VAD diet-fed mice. Parallel to this, a marked activation of the intestinal NF-*κ*B pathways and upstream proinflammatory machinery was also evident. After VAD diet-fed mice were treated with VADR diet, the inflammatory response in intestinal tissues was improved. VA has been previously shown to affect the progress of acute colitis by inhibiting NF-*κ*B activation [38]. The balance between pro- and anti-inflammatory mechanisms is affected by the commensal microbiota and bacterial spectrum [39, 40]. Thus, these data demonstrate that VAD stimulates the inflammatory response, which activates the NF-*κ*B pathway and increases the production of local proinflammatory cytokines, subsequently causing changes in intestinal microbiota.

Hence, we have found in the mouse model of dietary VA deficiency, the intestinal microbiota alterations were one of the factors that promoted glucose intolerance and islet dysfunction. The finding of this study indicated that therapeutic strategies targeting intestinal microbiota may be an effective treatment for prevention of VAD-associated islet dysfunction in the future.

## Figures and Tables

**Table 1:** Sequences of primers used for quantitative PCR.

Gene	Primer sequence(5′-3′)	
RAR*α*	F:CCATGTACGAGAGTGTGGAAGTC	R: CCTGGTGCGCTTTGCGA
RAR*β*	F: GATCCTGGATTTCTACACCG	R: CACTGACGCCATAGTGGTA
RXR*α*	F: CGCTCCTCAGGCAAACACTA	R: GGAGGATGCCGTCTTTCACA
RXR*β*	F: CTTCGGGAGAAGGTGTACGC	R: GGCAACACTTAGCAGGGTTC
LRAT	F: GCCTCCAAGACTGTCACGAA	R: AGTACAAGCTGGCCTTCGAC
CRBP1	F: GCTGAGCACTTTTCGGAACT	R: GGAGTTTGTCACCATCCCAG
RolDH	F: GCAAAGACTCGTCAGACCCA	R: GATCTCCTCCTGCATCACCG
*β*-Actin	F: AGGGAAATCGTGCGTGACAT	R: CGCAGCTCAGTAACAGTCCG

## Data Availability

The raw data supporting the conclusions of this manuscript will be made available by the authors for researchers who meet the criteria for access to confidential data.
